# Ebola Virus Outbreak Investigation, Sierra Leone, September 28–November 11, 2014 

**DOI:** 10.3201/eid2111.150582

**Published:** 2015-11

**Authors:** Hui-Jun Lu, Jun Qian, David Kargbo, Xiao-Guang Zhang, Fan Yang, Yi Hu, Yang Sun, Yu-Xi Cao, Yong-Qiang Deng, Hao-Xiang Su, Foday Dafae, Yu Sun, Cheng-Yu Wang, Wei-Min Nie, Chang-Qing Bai, Zhi-Ping Xia, Kun Liu, Brima Kargbo, George F. Gao, Jia-Fu Jiang

**Affiliations:** Key Laboratory of Jilin Province for Zoonosis Prevention and Control, Changchun, China (H.-J. Lu, J. Qian, Yang Sun, C.-Y. Wang, Z.-P. Xia);; Ministry of Health and Sanitation, Freetown, Sierra Leone (D. Kargbo, F. Dafae, B. Kargbo);; Chinese Center for Disease Control and Prevention, Beijing, China (X.-G. Zhang, Y.-X. Cao, G.-F. Gao);; Chinese Academy of Medical Sciences, Beijing (F. Yang, H.-X. Su);; China and Peking Union Medical College, Beijing (F. Yang, H.-X. Su);; State Key Laboratory of Pathogen and Biosecurity, Beijing (Y. Hu, Y.-Q. Deng, Yu Sun, K. Liu, J.-F. Jiang);; The No. 302 Hospital, Beijing (W.-M. Nie); The No. 307 Hospital, Beijing (C.-Q. Bai)

**Keywords:** Ebola virus disease, Ebola virus, Sierra Leone, western Africa, epidemiologic characteristics, clinical features, virus load, transmission, viruses, laboratory testing, investigation, epidemiology, epidemic, outbreak, control measures, treatment

## Abstract

During 2014–2015, an outbreak of Ebola virus disease (EVD) swept across parts of West Africa. The China Mobile Laboratory Testing Team was dispatched to support response efforts; during September 28–November 11, 2014, they conducted PCR testing on samples from 1,635 suspected EVD patients. Of those patients, 50.4% were positive, of whom 84.6% lived within a 3-km zone along main roads connecting rural towns and densely populated cities. The median time from symptom onset to testing was 5 days. At testing, 75.7% of the confirmed patients had fever, and 94.1% reported at least 1 gastrointestinal symptom; all symptoms, except rash and hemorrhage, were more frequent in confirmed than nonconfirmed patients. Virus loads were significantly higher in EVD patients with fever, diarrhea, fatigue, or headache. The case-fatality rate was lower among patients 15–44 years of age and with virus loads of <100,000 RNA copies/mL. These findings are key for optimizing EVD control and treatment measures.

## Abstract 2

Knowledge of epidemiologic, clinical, and viral features of the outbreak is critical for optimizing control and treatment measures.

Ebola virus disease (EVD) is a severe, frequently fatal illness. In March 2014, the largest EVD outbreak in history began spreading through parts of West Africa. As of June 21, 2015, a total of 27,443 cases, including 11,207 deaths, had been reported, of which 13,059 cases and 3,928 deaths were in Sierra Leone (*1*). Case numbers are believed to be underreported because they do not include many persons with clinically confirmed EVD who evaded laboratory confirmation and persons with suspected EVD who died and were buried without a confirmed diagnosis (*2*). This epidemic became an international public health emergency, and teams of public health experts continue to be deployed to affected areas to help with disease control efforts.

To support Sierra Leone and to respond to the World Health Organization (WHO) and United Nations’ appeals to help western Africa control the EVD epidemic, the China Mobile Laboratory Testing Team (CMLTT) was dispatched in September 2014 at the request of the Sierra Leone government (*3*). The team, equipped with medical experts who specialize in laboratory testing, epidemiology, clinical medicine, and nursing, worked at the Sierra Leone–China Friendship Hospital in Jui, a town in Western Area, Sierra Leone, ≈30 km southeast of Freetown (Figure 1; Technical Appendix Figure 1). The CMLTT was tasked with testing clinical samples for EVD; the samples were mainly collected from suspected EVD patients receiving care in Sierra Leone’s Western Area and Northern Province. All CMLTT activities were coordinated by an emergency operations center jointly established by the Sierra Leone Ministry of Health and Sanitation (MoHS) and WHO. We report the epidemiologic and clinical characteristics of a geographically distinct case series of live and deceased suspected EVD patients, from whom samples were collected and tested by the CMLTT.

**Figure 1:**
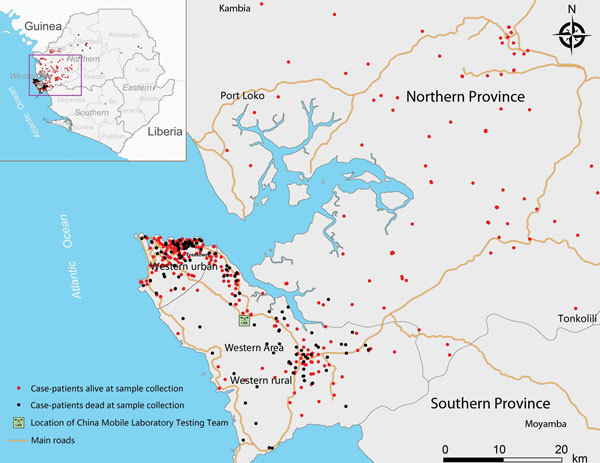
Geographic distribution of Ebola virus disease cases confirmed by the China Mobile Laboratory Testing Team (CMLTT), Sierra Leone, September 28–November 11, 2014. Inset shows the location of areas shown in the enlarged map. Western Area and parts of Northern Province and Southern Province are indicated on the enlarged map, as are rural and urban sections of Western Area.

## Material and Methods

### Study Design and Patients

The study included all suspected EVD patients (also called persons under investigation [PUI], per the WHO case definition at the time) from whom blood or oral swab samples were collected and sent to CMLTT for Ebola virus testing during September 28–November 11, 2014. A standardized WHO case investigation form was completed for each PUI by health care workers at the time of sample collection; the forms contained demographic information and information regarding signs and symptoms of disease, hospitalization, epidemiologic risk factors, and possible or known exposures to Ebola virus. For retrospective diagnosis of Ebola virus infection, we collected oral swab samples from deceased suspected EVD patients; information on age, sex, and address of previous residence were obtained from simple burial records for these persons. Using the case definition for disease surveillance developed by WHO, we defined confirmed EVD case-patients as persons (alive or dead) with suspected EVD whose samples were confirmed to be Ebola virus–positive by laboratory testing (*1*,*2*). For case-patients who were alive at sample collection, the definitive clinical outcomes were obtained at the end of December 2014 from a viral hemorrhagic fever database managed by the Sierra Leone MoHS.

### Laboratory Testing

Before use, blood and oral swab samples from PUIs were inactivated (62°C for 60 min) within the mobile Biosafety Level 3 laboratory as previously described (*4*). RNA was extracted from samples by using the QIAamp Viral RNA Mini Kit (QIAGEN, Germantown, MD, USA) according to the manufacturer’s instructions. Quantitative reverse transcription PCR targeting the glycoprotein gene of Ebola virus subtype Zaire was performed by using primer pairs 5′-TGGGCTGAAAAYTGCTACAATC-3′ and 5′-CTTTGTGMACATASCGGCAC-3′ and probe FAM-5′-CTACCAGCAGCGCCAGACGG-3′-TAMR as previously described (*5*). The cycle threshold cutoff value was 36. For quantification, virus loads were estimated as Ebola virus RNA copies per milliliter (Technical Appendix).

### Ethical Considerations

This work was conducted as part of the surveillance and public health response to contain the EVD outbreak in Sierra Leone. Activities were coordinated by the emergency operations center established by the Sierra Leone MoHS and WHO. All data obtained from this work belong to the Sierra Leone MoHS and were shared with CMLTT for reporting. The data were submitted to the Sierra Leone National Ethics and Scientific Research Committee. All information regarding individual persons has been anonymized in this report.

### Data Analyses

Each confirmed case was georeferenced and linked to a digital map of Sierra Leone (http://www.mapmakerdata.co.uk.s3-website-eu-west-1.amazonaws.com/library/stacks/Africa/Sierra%20Leone/) according to the residential address of the case-patient by using ArcGIS 9.2 software (Esri, Redlands, CA, USA). We then conducted a proximity analysis of confirmed cases in relation to the main transportation routes. We identified epidemiologic and clinical data for each case-patient by extracting the necessary information from the case investigation form. The case-fatality rate was calculated as the percentage of persons who died among the confirmed EVD case-patients with a known definitive clinical outcome; outcome information was attained from the viral hemorrhagic fever database that was updated by Sierra Leone MoHS and WHO. Descriptive statistics to do with measures of central tendency and dispersion, such as mean, mode, and median, were calculated for all variables. Continuous variables were summarized as median, mean ± SD, and range; categorical variables were summarized as frequencies and proportions. To estimate the differences between groups, we used Student *t* test, χ^2^ test, or Fisher exact test, as appropriate. A 2-sided p<0.05 was considered statistically significant. All statistical analyses were conducted by using SAS software version 9.3 (SAS Institute, Inc., Cary, NC, USA).

## Results

### Patients

During September 28–November 11, 2014, a total of 1,635 samples from PUIs were sent to CMLTT at the Sierra Leone–China Friendship Hospital in Jui for EVD testing. A total of 824 (50.4%) samples were Ebola virus–positive; details regarding the samples, results, and case-patients are presented in Technical Appendix Figure 2. These 824 confirmed cases represented 33.3% of 2,471 total confirmed cases reported in Sierra Leone during the study period (Technical Appendix Figure 3).

### Epidemiologic Characteristics

The numbers of samples received by CMLTT and the rate of positive samples varied from day to day (Technical Appendix Figure 4); however, the average percentage of positive samples during the last 10 days of testing (November 1–11, 2014) was significantly lower than that during September 28–October 31, 2014 (41.2% vs. 57.0%, respectively; p<0.001). A comparison of the weekly numbers of tested samples and positivity rates for case-patients who were alive and those who were deceased showed similar temporal variations (Technical Appendix Figure 5).

The median age of confirmed EVD case-patients was 26 years (range 2 days to 99 years); 7.1% of the patients were <5 years of age (Technical Appendix Table 1). Cases occurred in 9 districts of Sierra Leone, mainly in Western Area around the mobile Biosafety Level 3 laboratory catchment area. Most (84.6%) confirmed cases were distributed within a 3-km zone along the main roads that connect rural and urban areas (Figure 1).

The sex distribution for live and deceased case-patients (as defined as the outcome at time of testing) was similar (p = 0.52), and deceased case-patients were significantly older than live case-patients (p = 0.004) (Technical Appendix Table 1). Oral swab samples were tested for 404 deceased persons (391 from Western Area, 12 from Northern Province, and 1 from Eastern Province); however, they could not be included in further analyses because only simple demographic information on age, sex, and address of residence was available in the patients’ burial records.

**Table 1:** Demographic and clinical characteristics for suspected Ebola virus disease patients, Sierra Leone, September 28–November 11, 2014*

Characteristics	No. (%) patients with positive RT-PCR results			No. (%) patients with negative RT-PCR results, n = 451
	All patients, n = 563	Patients who died, n = 328	Patients who recovered, n = 235	
Demographic characteristic				
Sex				
Female	266 (47.2)	156 (47.6)	110 (46.8)	219 (48.6)
Male	297(52.8)	172(52.4)	125(53.2)	232(51.4)
Age, y, group†				
0–5	30 (5.3)	18 (5.6)	12 (5.1)	53 (12.0)
6–14	105 (18.7)	68 (21.0)	37 (15.8)	59 (13.4)
15–44	341 (61.1)	179 (55.2)	162 (69.2)	261 (59.3)
>45	82 (14.7)	59 (18.2)	23 (9.8)	67 (15.2)
Location of residence				
Western Area				
Rural areas	198 (35.2)	107 (32.6)	91 (38.7)	137 (30.4)
Urban areas	140 (24.9)	71 (21.6)	69 (29.4)	177 (39.2)
Northern Province				
Port Loko District	191 (33.9)	131 (39.9)	60 (25.5)	71 (15.7)
Kambia District	21 (3.7)	13 (4.0)	8 (3.4)	27 (6.0)
Bombali District	10 (1.8)	5 (1.5)	5 (2.1)	23 (5.1)
Koinadugu District	2 (0.4)	1 (0.3)	1 (0.4)	5 (1.1)
Tonkolili District	0	0	0	9 (1.2)
Southern Province				
Bo Town	1 (0.2)	0	1 (0.4)	0
Bonthe District	0	0	0	1 (0.1)
Signs and symptoms				
Fatigue	464 (84.4)	272 (82.9)	192 (81.7)	196 (43.5)
Anorexia	467 (82.9)	278 (84.8)	189 (80.4)	208 (46.1)
Fever	426 (75.7)	260 (79.3)	166 (70.6)	210 (46.6)
Vomiting or nausea	354 (62.9)	202 (61.6)	152 (64.7)	112 (24.8)
Headache	354 (62.9)	209 (63.7)	145 (61.7)	195 (43.2)
Diarrhea	349 (62.0)	207 (63.1)	142 (60.4)	103 (22.8)
Joint pain	319 (56.7)	186 (56.7)	133 (56.6)	174 (38.6)
Abdominal pain	317 (56.3)	184 (56.1)	133 (56.6)	130 (28.8)
Muscle pain	305 (54.2)	183 (55.8)	122 (51.9)	137 (30.4)
Chest pain	226 (40.1)	123 (37.5)	103 (43.8)	96 (21.3)
Cough	212 (37.7)	113 (34.5)	99 (42.1)	101 (22.4)
Difficulty breathing	191 (33.9)	110 (33.5)	81 (35.4)	88 (19.5)
Difficulty swallowing	164 (29.1)	95 (29.0)	69 (29.4)	56 (12.4)
Conjunctivitis	161 (28.6)	95 (29.0)	66 (28.1)	34 (7.5)
Confused	151 (26.8)	88 (26.8)	63 (26.8)	50 (11.1)
Sore throat	117 (20.8)	66 (20.1)	51 (21.7)	39 (8.6)
Jaundice	102 (18.1)	63 (19.2)	39 (16.6)	42 (9.3)
Hiccups	95 (16.9)	56 (17.1)	39 (16.6)	20 (4.4)
Pain behind eyes	55 (9.8)	34 (10.4)	21 (8.9)	13 (2.9)
Skin Rash	45 (8.0)	25 (7.6)	20 (8.5)	23 (5.1)
Coma	27 (4.8)	15 (4.6)	12 (5.1)	10 (2.2)
Hemorrhage	6 (1.1)	6 (1.8)	0	7 (1.6)
Virus load, mean± SD‡	363,078 ±28	436,515 ±26	288,403 ±30	NA

*NA, not applicable; RT-PCR, reverse transcription PCR. †Age was not known for 5 patients with confirmed disease and 11 patients with negative test results. ‡Virus loads represent RNA copies.

### Clinical Characteristics

Of 666 confirmed EVD patients who were alive when samples were collected, 606 had provided information on clinical manifestations of the disease on their case investigation forms, and 563 had a known clinical outcome (Technical Appendix Figure 2). The most commonly reported symptoms were fatigue, anorexia, fever, headache, vomiting or nausea, diarrhea, abdominal pain, joint pain, and muscle pain (Table 1). Of these 563 case-patients, 530 (94.1%) reported as least 1 gastrointestinal symptom (anorexia, nausea, vomiting, diarrhea, abdominal pain, or hiccups), and 426 (75.7%) had fever. Hemorrhage (i.e., hemoptysis, bleeding from the gums and nose, hematochezia, hematuria, bleeding at injection sites, and vaginal bleeding) was observed in 6 (1.1%) patients. All signs and symptoms, except skin rash and hemorrhage, were more frequently observed in patients with confirmed EVD than in those with negative test results (p<0.05) (Table 1). The median time from symptom onset to seeking care at an Ebola health facility (i.e., holding or treatment center) for EVD testing was 5.0 days (interquartile range 3.0–7.0 days) (Technical Appendix Figure 6).

**Figure 2:**
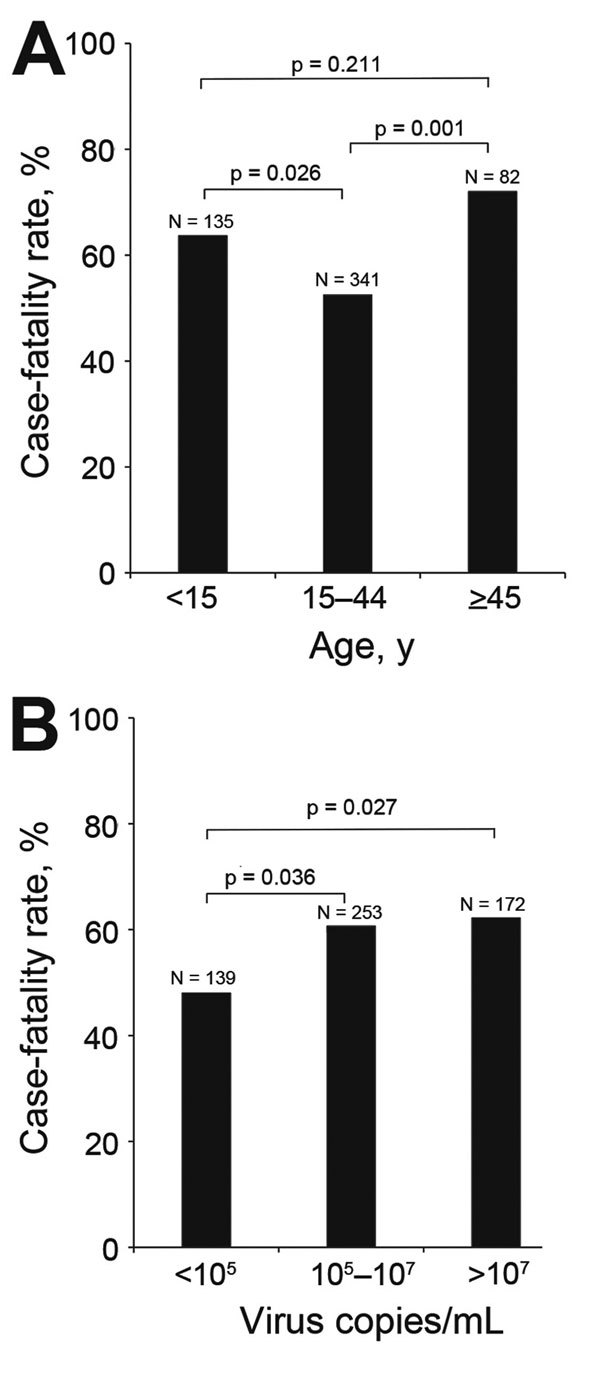
Case-fatality rates among patients with Ebola virus disease, Sierra Leone, September 28–November 11, 2014. A) Rates among different age groups. B) Rates among persons with different virus loads. The total number of patients in each group is shown at the top of the respective bar.

Among the 563 case-patients, the overall case-fatality rate was 67.4%. Case-fatality rates for persons >45 years of age (72.0%) and persons <15 years of age (63.7%) were significantly higher than that for persons 15–44 years of age (52.5%) (p = 0.001 and p = 0.026, respectively) (Figure 2, panel A). The case-fatality rate for case-patients with fever was significantly higher than that for case-patients without fever (61.0% [260/426 patients]) vs. 49.6% [68/137 patients]; p = 0.019).

### Virus Loads

**Figure 3:**
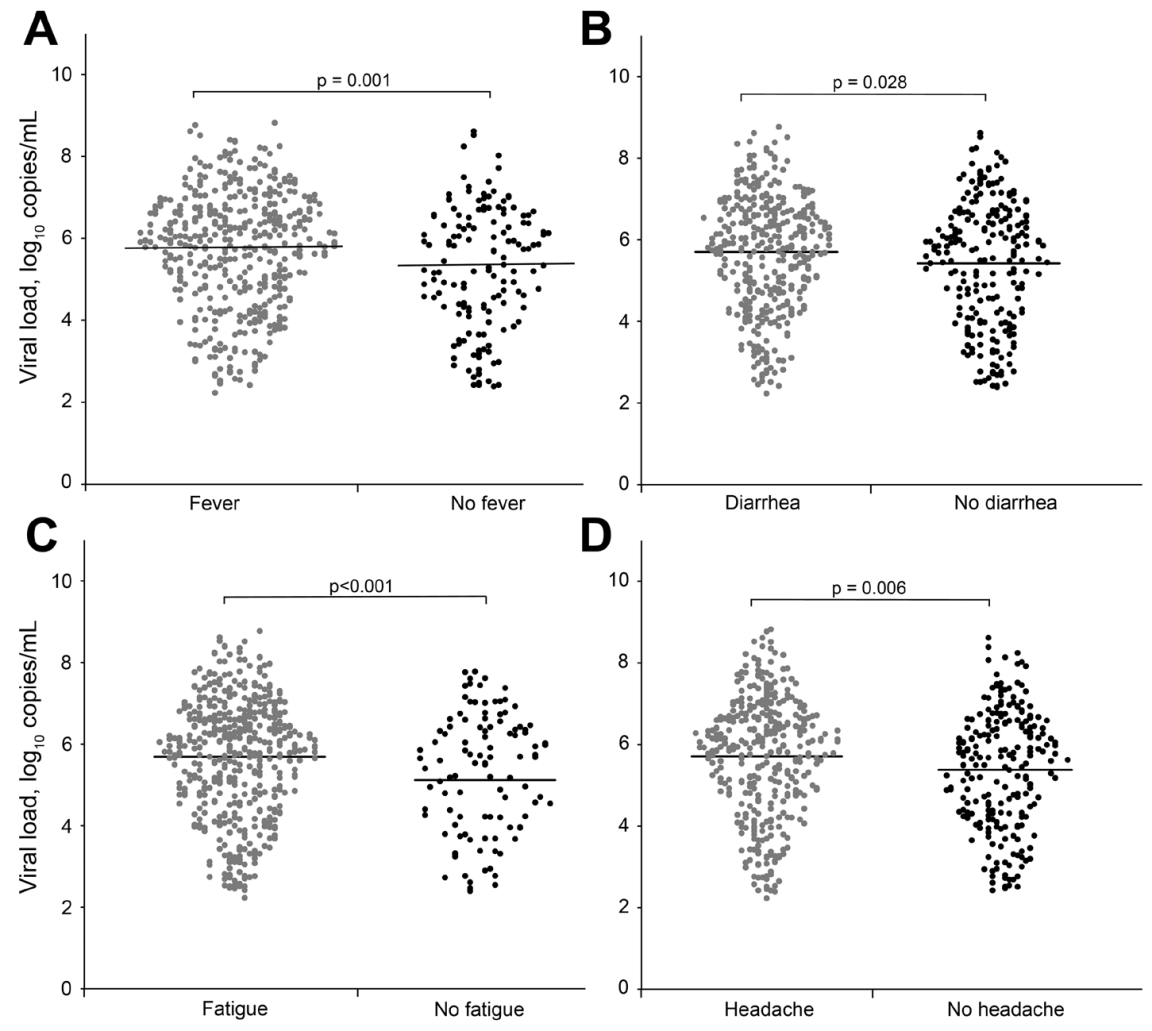
Comparison of virus loads for patients with Ebola virus disease with and without fever (A), diarrhea (B), fatigue (C), or headache (D). Dots represent the log-transformed virus loads in patients with and without each symptom. The horizontal line in each panel indicates the mean value of log-transformed virus loads for each group.

For comparison, we quantified and log-transformed the Ebola virus load (RNA copies/mL) for each patient with confirmed EVD. The mean virus load for EVD patients at admission to an Ebola health facility (i.e., the day of testing) varied depending on the time between the onset of signs and symptoms and admission. Mean virus load continued to increase for patients tested 1–3 days after symptom onset; values peaked at 3–7 days, began decreasing at 7–14 days, and continued decreasing thereafter (Technical Appendix Figure 7). Virus loads for case-patients with fever, diarrhea, fatigue, and headache were significantly higher than those for case-patients without these symptoms (Figure 3). Case-patients with 10^5^–10^7^ or >10^7^ viral RNA copies/mL had higher case-fatality rates than did case-patients with <10^5^ viral RNA copies/mL (p = 0.036 and p = 0.027, respectively) (Figure 2, panel B).

## Discussion

During September 28–November 11, 2014, we confirmed that a total of 824 persons in Sierra Leone were positive for EVD; this number represents one third of the reported cases in the country during this period (*6*). Most (84.6%) case-patients identified in this study resided within a 3-km zone along the main roads of Sierra Leone, which are vital connections between rural towns and densely populated cities. This finding suggests that epidemic dispersal of Ebola virus is promoted when infectious persons live in close proximity to main roads. These roads provide a convenient source of transportation for persons traveling to Ebola health facilities, which may have enabled the rapid and extensive spread of Ebola virus infection in Sierra Leone through person-to-person contact. In contrast, the simultaneous EVD outbreak in the Democratic Republic of Congo was much smaller, probably because it occurred in remote forested areas where person-to-person contact outside the local population may be more limited because access to transportation is limited (*7*).

Of note, 39.1% of swab samples collected from deceased persons were positive for Ebola virus RNA. The prompt confirmation of Ebola virus infection in dead persons can contribute to a reduction of virus transmission during funerals because safe burial practices are required in affected areas once a diagnosis of EVD is made (*1*). To decrease the transmission of Ebola virus through unsafe burial practices, samples should be collected from and a diagnosis should be determined for persons who die from unknown causes (*8*).

In our study, the most common symptoms for persons with confirmed EVD were fatigue, anorexia, fever, vomiting or nausea, headache, diarrhea, joint pain, abdominal pain, and muscle pain; these findings are comparable to those from other studies in Sierra Leone (*9*–*11*). Of the patients in our study, 94.1% had at least 1 gastrointestinal symptom; nausea, vomiting, and diarrhea were common and caused severe dehydration and electrolyte abnormalities that subsequently led to circulatory collapse and death. The high frequency of gastrointestinal symptoms further supports the proposal for administration of intravenous fluids and electrolytes in the treatment of EVD (*12*). Signs and symptoms, including the low frequency of hemorrhagic signs, for patients in our study were similar to those for contemporary case-patients in studies in other affected countries (Table 1; Technical Appendix Table 2) (*2*,*11*,*13*). However, our results indicate that persons infected during this outbreak showed a lower frequency of the primary clinical symptoms than did persons infected with a different Ebola virus strain (Bundibugyo) during an outbreak in Uganda in 2007 (Technical Appendix Table 2) (*13*). 

In our study, 75.7% (426/563) of the confirmed case-patients had a fever when their specimens were collected and tested for Ebola virus. These findings are similar to those of our colleagues, Qin et al. (*14*), who found that 18.0% (11/61) of patients in the Sierra Leone–China Friendship Hospital did not have a fever on the day of admission. During these studies, the field case definition for fever was temperature >38.0°C at the time of assessment or a history of fever. This finding implies that persons with suspected EVD but without fever may still be infective.

Our analysis showed that, except skin rash and hemorrhage, all clinical symptoms that were surveyed in the case investigation form were more frequently observed in patients with than those without confirmed EVD. This finding suggests that the case definition in use at the time was appropriate for this outbreak. In our study, the case-fatality rate was 67.4% among confirmed EVD case-patients who were alive when samples were obtained; this rate is comparable to those reported for Sierra Leone by the WHO (*2*). Other studies on EVD in Sierra Leone reported a 73.6% (64/87) case-fatality rate for cases during May 25–June 18, 2014, in Eastern Province (*9*); a 31.5% (183/581) case-fatality rate during September 20–December 7, 2014, at Hastings Treatment Center in Western Area (*10*); and a 24.6% case-fatality rate (1,169 confirmed cumulative deaths among 4,744 confirmed cases) reported by the Sierra Leone MoHS during May 23–November 11, 2014 (*6*). Patients 15–44 years of age had a lower case-fatality rate than older and younger patients. This association of age with the death rate was similar to that observed in the early stage of the EVD outbreak in West Africa (*2*). Of note, in our study, the case-fatality rate for patients <15 years of age was relatively high compared with that reported in Eastern Province (*10*). These findings indicate that older patients and children <15 years of age should receive more medical attention to reduce their higher case-fatality rate and that investigations are needed to determine why EVD case-fatality rates differ by patient age.

In agreement with findings by Schieffelin et al. (*9*), we found that a low virus load at admission to a treatment facility was associated with a better outcome. However, those results might have been different had we used a cutoff value of 10^5^ in 3 categories, similar to the cycle threshold value of 25 that was described in a recent article by Fitzpatrick et al. (*15*). We also found that patients with fever, diarrhea, fatigue, or headache had virus loads that were significantly higher than those for patients without these symptoms; this finding is consistent with those from other studies (*9*,*15*).

The number of confirmed cases in our analysis was quite large, accounting for one third of the cases reported in Sierra Leone during the study period. Nevertheless, our study had several limitations. First, the inclusion of confirmed EVD patients whose samples were sent to CMLTT for Ebola virus testing was subject to selection bias because the samples collected from PUIs were delivered to laboratories in a haphazard manner. Second, the purpose of our testing was to intensify the outbreak response efforts, not to conduct surveillance or accurately ascertain the prevalence of disease. Last, information on many case investigation forms was incomplete because the data were collected in the context of response operations and used for clinical care, contact tracing, and transmission prevention rather than for a rigorous epidemiologic survey. Because of these limitations, our results should be interpreted with discretion. 

These findings provide key information for informing public health decision-making during Ebola virus outbreaks. EVD control measures and treatment methods should be optimized according to the transmission, clinical, and viral features specific to each outbreak.

Technical Appendix Additional information regarding an Ebola outbreak investigation in Sierra Leone by the China Mobile Laboratory Testing Team.
