# Supplementary material for: Ebola Virus Outbreak Investigation, Sierra Leone, September 28–November 11, 2014
Source: Emerg Infect Dis. 2015 Nov;21(11):1921–7. doi: 10.3201/eid2111.150582 (PMC4622249; doi:10.3201/eid2111.150582)
Supplement: Technical Appendix — Additional information regarding an Ebola outbreak investigation in Sierra Leone by the China Mobile Laboratory Testing Team. [file 15-0582-Techapp-s1.pdf]

Article DOI: http://dx.doi.org/10.3201/eid2111.150582

Ebola Virus Outbreak Investigation, Sierra Leone, September 28–November 11, 2014

Technical Appendix

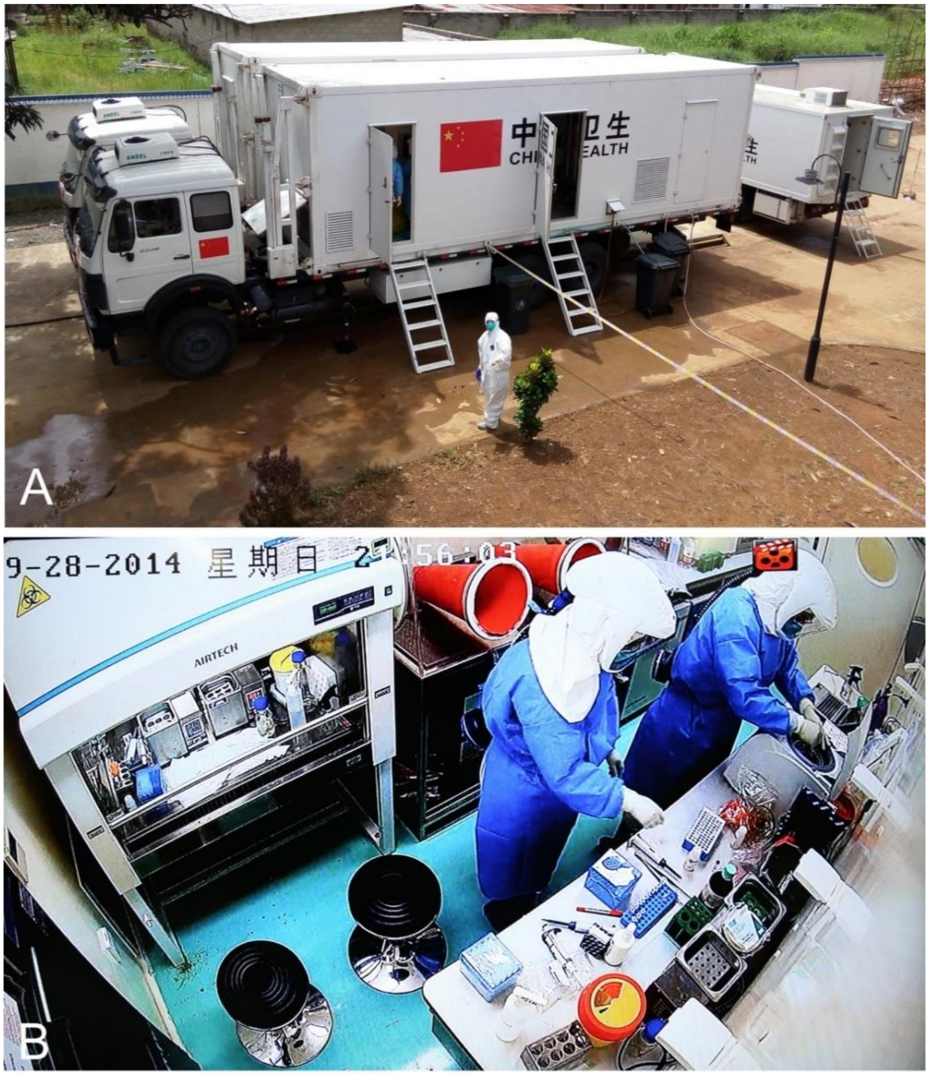

Technical Appendix Figure 1. Pictures of the China mobile BSL-3 laboratory at Sierra Leone–China Friendship Hospital. The hospital is located in Jui Town, Sierra Leone, ≈30 Km southeast of Freetown. A)

The China mobile Biosafety Level 3 laboratory in the yard of Sierra Leone–China Friendship Hospital. B) China Mobile Laboratory Testing Team members working inside the laboratory.

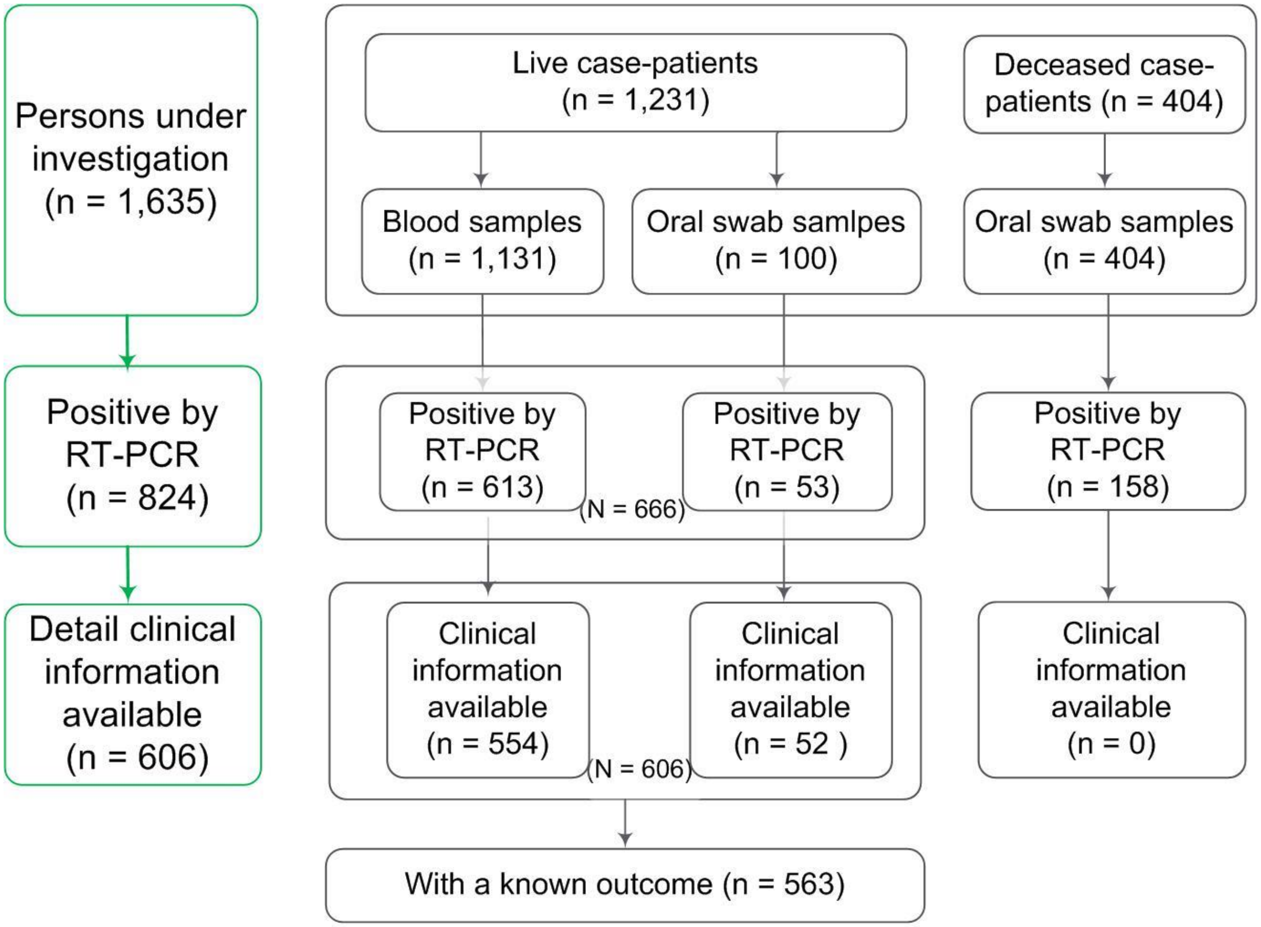

Technical Appendix Figure 2. The numbers of persons under investigation and laboratory-confirmed cases of Ebola virus disease for each analysis in this study in Sierra Leone, September 28–November 11, 2014. The term live case-patients refers to patients who were alive at the time of sample collection; deceased case-patients refers to patients who were dead at the time of sample collection.

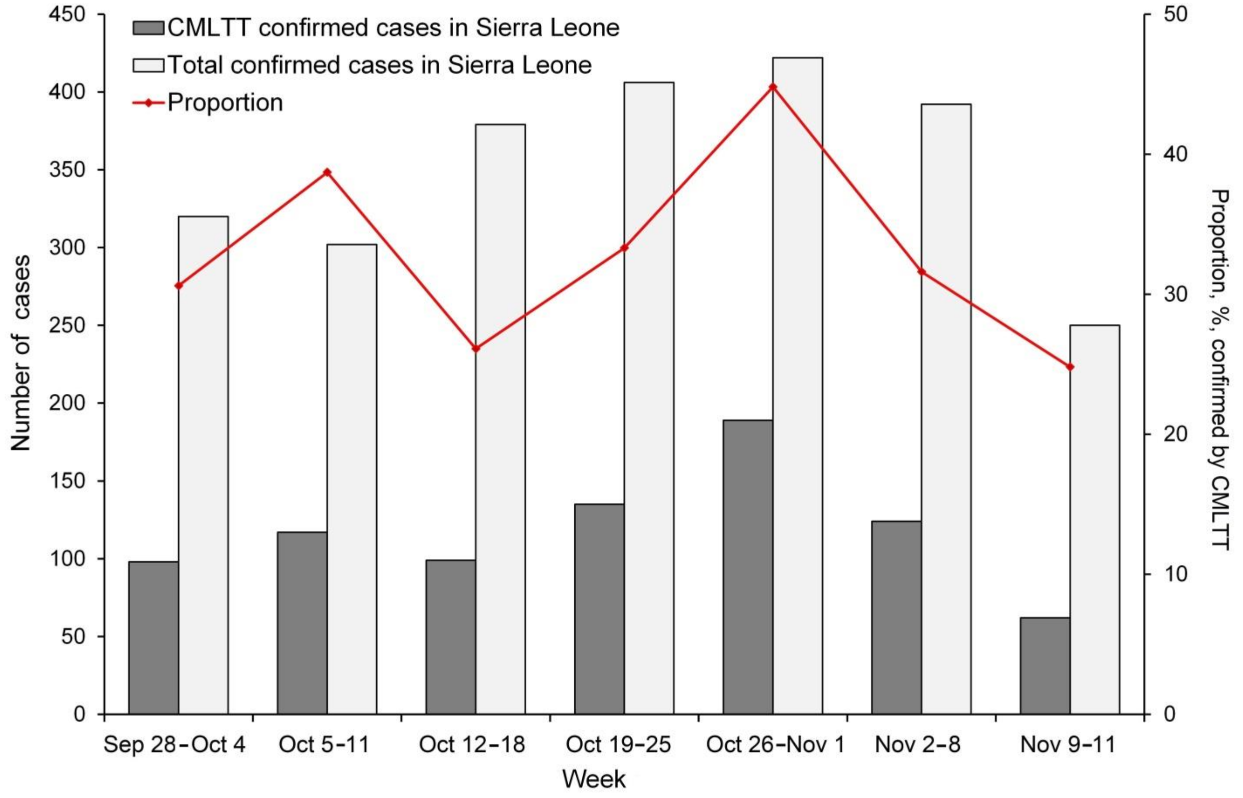

Technical Appendix Figure 3. Comparison of the weekly number of Ebola virus disease cases confirmed in Sierra Leone by the China Mobile Laboratory Testing Team (CMLTT) and the weekly number reported in the daily Ebola situation report prepared by the Ministry of Health and Sanitation of Sierra Leone (http://health.gov.sl/?page_id=583), September 28–November 11, 2014. The red line represents the proportion of all reported cases that were confirmed by CMLTT.

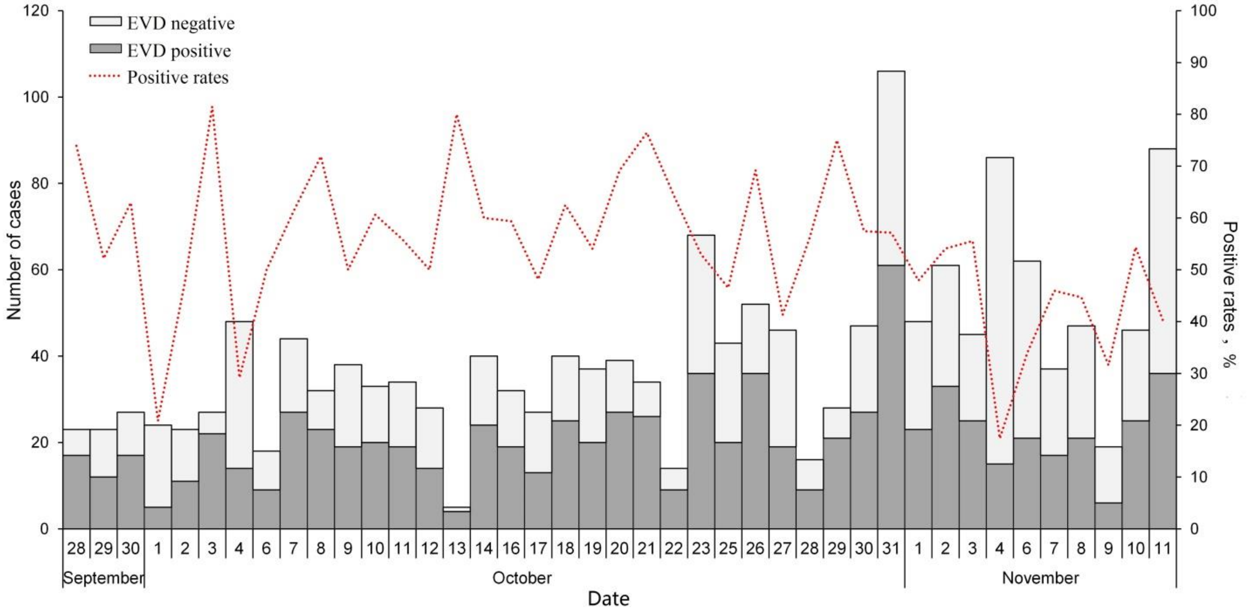

Technical Appendix Figure 4. Temporal distribution of persons under investigation for Ebola virus disease who were tested by the China Mobile Laboratory Testing Team (CMLTT), Sierra Leone, September 28–November 11, 2014. The CMLTT maintained the Biosafety Level 3 laboratory and did not perform RT-PCR assays on October 5, 15, and 24 and November 5, 2014.

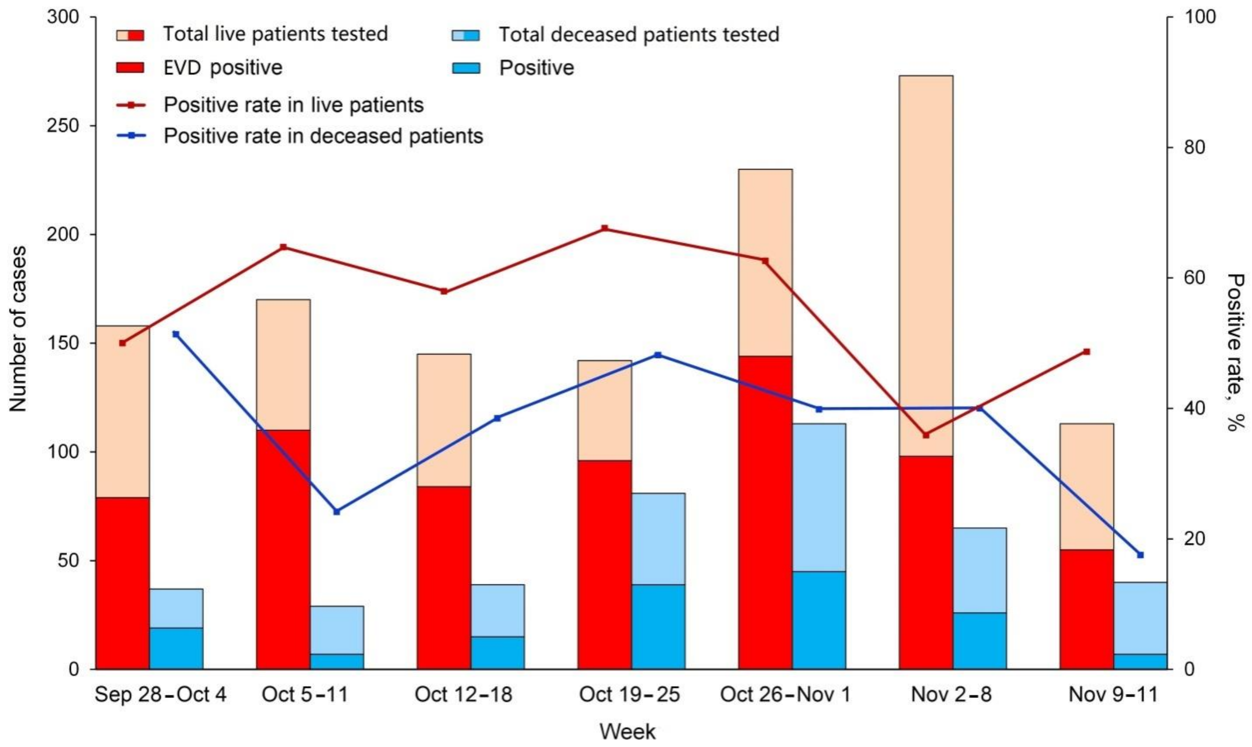

Technical Appendix Figure 5. Comparison of the number of tested samples and Ebola virus positivity rates between alive and deceased persons tested by the China Mobile Laboratory Testing Team, Sierra Leone, September 28–November 11, 2014. The red bars represent the weekly numbers of live persons positive for Ebola virus. The mango bars represent the weekly numbers of live persons negative for Ebola virus. The blue bars represent the weekly numbers of deceased persons positive for Ebola virus. The cyan bars represent the weekly numbers of deceased persons negative for Ebola virus. The red and blue lines represent the positive rates in for live and deceased patients, respectively.

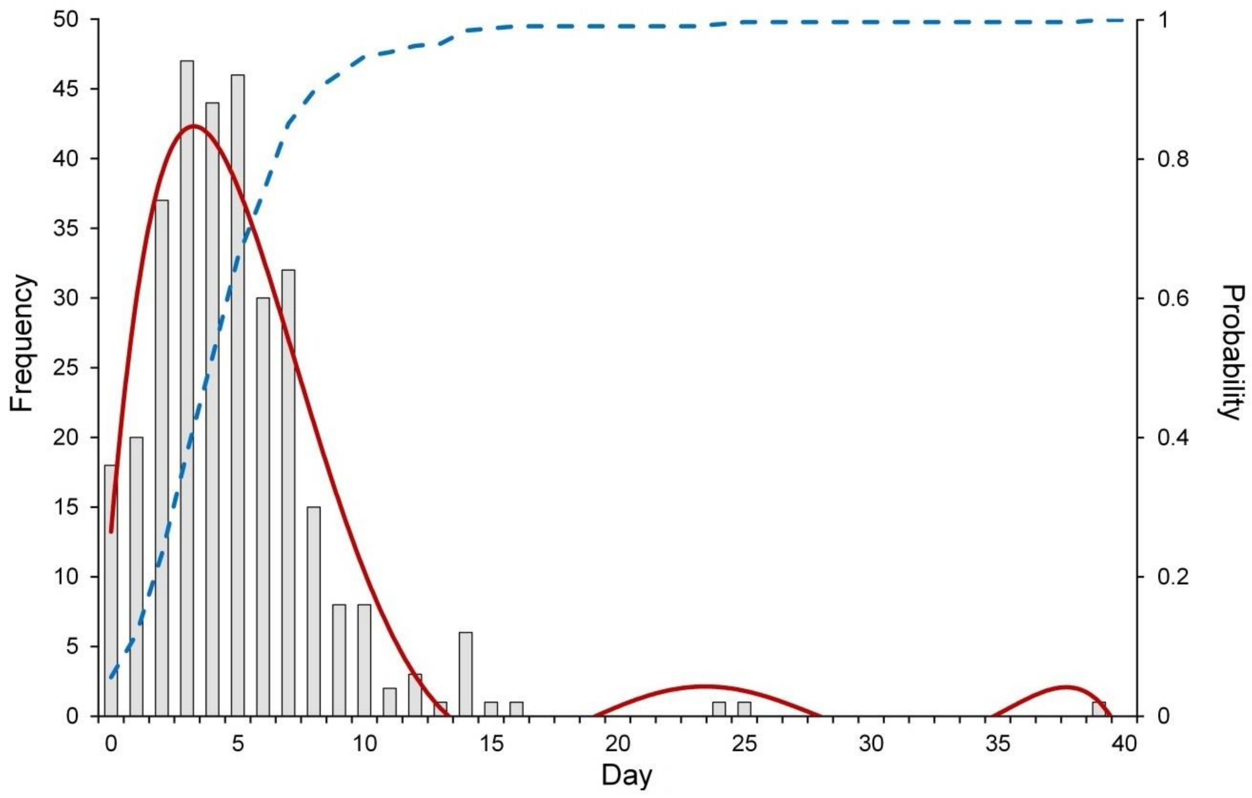

Technical Appendix Figure 6. Interval from symptom onset to the time of seeking medical care at an Ebola holding or treatment center. The gray bars represent the frequency of observed interval days. The red curve represents best fitted function for the frequency of observed interval, and the blue curve represents cumulative distribution for the interval period.

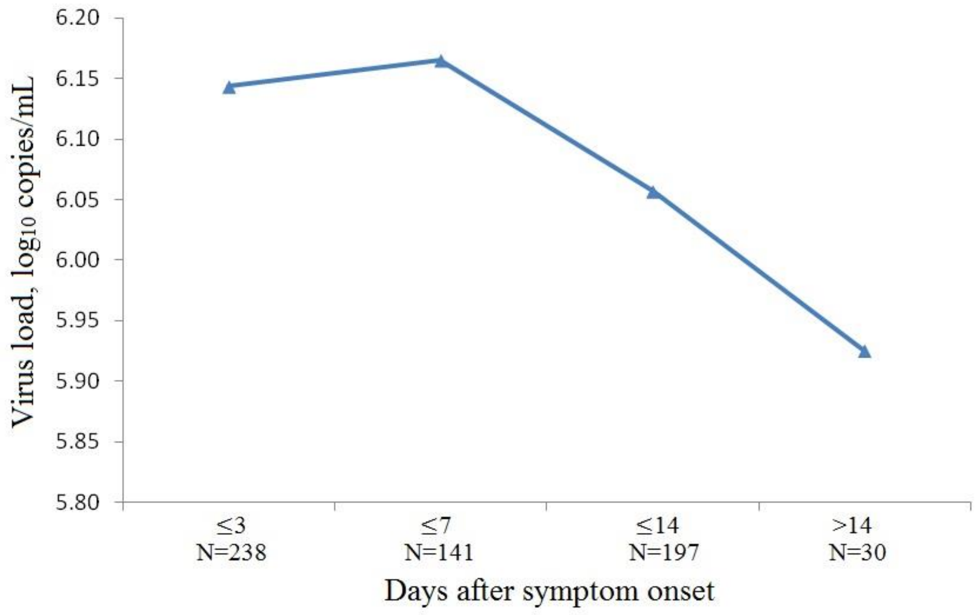

Technical Appendix Figure 7. Virus loads on different days after symptom onset for 606 confirmed Ebola virus disease case-patients who had available clinical information, Sierra Leone, September 28 to November 11, 2014.

Technical Appendix Table 1. Demographic characteristics for 824 persons with confirmed Ebola virus disease when they sought medical care at a holding or treatment center, Sierra Leone, September 28–November 11, 2014*

Characteristics	Live case-patients, n = 66	Deceased case-patients, n = 158	Total confirmed patients, n = 824
Sex†			
F	316 (48.5)	65 (45.4)	381 (47.9)
M	336 (51.5)	78 (54.6)	414 (52.1)
Mean age, y, ± SD‡	27.6 ± 16.1	33.0 ± 20.1	28.6 ± 16.9
Median age, y (range)	25.0 (0.1–99.0)	33.0 (0.3–92.0)	26.0 (0.1–99.0)
Age group, y			
0–5	35 (5.5)	20 (14.8)	55 (7.1)
6–14	112 (17.5)	4 (3.0)	116 (14.9)
15–44	395 (61.6)	75 (55.6)	470 (60.6)
≥45	99 (15.4)	36 (26.7)	135 (17.4)
Location of residence			
Southern Province			
Bo Town	1 (0.2)	0	1 (0.1)
Moyamba District	1 (0.2)	0	1 (0.1)
Northern Province			

Characteristics	Live case-patients, n = 66	Deceased case-patients, n = 158	Total confirmed patients, n = 824
Bombali District	11 (1.7)	6 (3.8)	17 (2.1)
Kambia District	31 (4.7)	0	31 (3.8)
Koinadugu District	2 (0.3)	1 (0.6)	3 (0.4)
Port Loko District	213 (32.0)	0	213 (25.9)
Tonkolili District	1 (0.2)	0	1 (0.1)
Western Area			
Urban	176 (26.4)	65 (41.1)	241 (29.2)
Rural	230 (34.5)	86 (54.4)	316 (38.3)

*Data are no. (%) unless otherwise stated in the first column.

†Information on sex was available for 652 live case-patients and 143 deceased case-patients.

‡Information on age was available for 641 live case-patients and 135 deceased case-patients. The deceased case-patients were significantly older than live patients (p = 0.004).

Technical Appendix Table 2. Summary of demographic characteristics and signs and symptoms for persons in Sierra Leone with confirmed Ebola virus disease and a known outcome and for previously reported Ebola virus disease case-patients in Africa*

	No. (%) patients from this study, Sierra Leone, September 28–November 11, 2014			No. (%) patients from Sierra Leone, May 25–June 18, 2014 (*1*)			No. (%) patients from Uganda, August–November 2007 (*2*)			No. (%) patients from western Africa, March 23–September 14, 2014 (*3*),		
Variable	All, n = 563	Died, n = 328	Recovered, n = 235	All, n = 87	Died, n = 64	Recovered, n = 23	All, n = 43	Died, n = 26	Recovered, n = 17	All	Died	Recovered
Demographic characteristics												
Sex												
F	266 (47.2)	156 (47.6)	110 (46.8)	50 (57)	37 (58)	13 (57)	19 (44)	10 (38)	9 (53)	730 (51.6)	541 (51.2)	189 (52.6)
Age group, y†												
0–14	135 (24.2)	86 (26.5)	49 (20.9)	21 (24)	13 (20)	8 (35)				190 (13.8)	145 (14.2)	45 (12.6)
15–44	341 (61.1)	179 (55.2)	162 (69.2)	49 (56)	35 (55)	14 (61)				838 (60.8)	577 (56.5)	261 (73.1)
≥45	82 (14.7)	59 (18.2)	23 (9.8)	16 (18)	15 (23)	1 (4)				350 (25.4)	299 (29.3)	51 (14.3)
Location of residence							NA	NA	NA	NA	NA	NA
Western Area												
Rural	198 (35.2)	107 (32.6)	91 (38.7)									
Urban	140 (24.9)	71 (21.6)	69 (29.4)									
Northern Province												
Port Loko District	191 (33.9)	131 (39.9)	60 (25.5)									
Kambia District	21 (3.7)	13 (4.0)	8 (3.4)									
Bombali District	10 (1.8)	5 (1.5)	5 (2.1)									
Koinadugu District	2 (0.4)	1 (0.3)	1 (0.4)									
Southern Province												
Bo Town	1 (0.2)	0 (0.0)	1 (0.4)									
Eastern Province												
Kailahun District				91 (92)	59 (92)	21 (91)						
Kenema District				4 (4)	4 (6)	0 (0)						
Other in Sierra Leone					1 (2)	2 (9)						
Symptoms‡												
Fever	426 (75.7)	260 (79.3)	166 (70.6)	31 (89)	25 (89)	6 (86)	42 (100)	26 (100)	16 (100)	1,002 (87.1)	746 (88.2)	256 (83.9)
Vomit/nausea	354 (62.9)	202 (61.6)	152 (64.7)	12 (34)	12 (43)	0 (0)	37 (90)	24 (92)	13 (87)	753 (67.6)	566 (69.4)	187 (62.8)
Diarrhea	349 (62.0)	207 (63.1)	142 (60.4)	18 (51)	17 (61)	1 (14)	37 (90)	24 (92)	13 (87)	721 (65.6)	555 (68.3)	166 (58.0)
Fatigue	464 (84.4)	272 (82.9)	192 (81.7)	23 (66)	22 (79)	1 (14)	36 (97)	22 (96)	14 (100)	866 (76.4)	633 (76.4)	233 (76.6)
Anorexia	467 (82.9)	278 (84.8)	189 (80.4)				31 (86)	19/23 (83)	12/15 (80)	681 (64.5)	498 (64.0)	183 (66.1)
Abdominal pain	317 (56.3)	184 (56.1)	133 (56.6)	14 (40)	13 (46)	1 (14)	36 (90)	23/26 (88)	13/14 (93)	439 (44.3)	311 (43.5)	128 (46.2)
Muscle pain	305 (54.2)	183 (55.8)	122 (51.9)				31 (86)	19/23 (83)	12/14 (86)	385 (38.9)	293 (40.2)	92 (35.1)
Joint pain	319 (56.7)	186 (56.7)	133 (56.6)							374 (39.4)	283 (40.7)	91 (35.7)
Headache	354 (62.9)	209 (63.7)	145 (61.7)	28 (80)	23 (82)	5 (71)	35 (88)	21/25 (84)	14/15 (93)	553 (53.4)	407 (53.8)	146 (52.5)

Chest pain	226 (40.1)	123 (37.5)	103 (43.8)							254 (37.0)	196 (40.2)	58 (29.3)
Cough	212 (37.7)	113 (34.5)	99 (42.1)	7 (20)	6 (21)	1 (14)				194(29.6)	150 (32.5)	44 (22.8)
Difficulty breathing	191 (33.9)	110 (33.5)	81 (35.4)				14 (38)	6/23 (26)	8/14 (57)	155 (23.3)	123 (26.1)	32 (16.6)
Difficulty swallowing	164 (29.1)	95 (29.0)	69 (29.4)				16 (42)	10/23 (43)	6/15 (60)	169 (32.9)	138 (36.8)	31 (22.3)
Sore throat	117 (20.8)	66 (20.1)	51 (21.7)	12 (34)	11 (39)	1 (14)				102 (21.8)	82 (24.2)	20 (15.6)
Jaundice	102 (18.1)	63 (19.2)	39 (16.6)							65 (10.4)	52 (11.7)	13 (7.1)
Skin Rash	45 (8.0)	25 (7.6)	20 (8.5)	1(3)	1 (4)	0 (0)	14 (34)	9/26 (35)	5/15 (33)	37 (5.8)	30 (6.6)	7(3.7)
Conjunctivitis	161 (28.6)	95 (29.0)	66 (28.1)	11 (31)	10 (36)	1 (14)				137 (20.8)	109 (23.4)	28 (14.5)
Hiccups	95 (16.9)	56 (17.1)	39 (16.6)				10 (26)	4/23 (17)	6/15 (40)	108 (11.4)	91 (13.0)	17 (6.9)
Pain behind eyes	55 (9.8)	34 (10.4)	21 (8.9)							48 (7.7)	39 (8.9)	9 (4.9)
Coma	27 (4.8)	15 (4.6)	12 (5.1)							37 (5.9)	34 (7.6)	3 (1.6)
Confusion	151 (26.8)	88 (26.8)	63 (26.8)	7 (20)	7 (25)	0 (0)				84 (13.3)	68 (15.2)	16 (8.6)
Hemorrhage	6 (1.1)	6 (1.8)	0 (0)				20 (47)	11/26 (42)	9/17 (53)	168 (18.0)	140 (20.2)	28 (11.7)
Virus load												
Mean ± SD	363,078 ± 3	436,515 ± 36	288,403 ± 8									
$<10^5$	184 (32.7)	107 (32.6)	77 (32.8)		5 (11)	10 (56)						
10^5–10^7	286 (50.8)	163 (49.7)	123 (52.3)		23 (51)	7 (39)						
$>10^7$	93 (16.5)	58 (17.7)	35 (14.9)		17 (38)	1 (5)						

*NA, not applicable.

†Age was not available for 5 patients.

‡Only 44 of the patients reported during May 25–June 18 in Sierra Leone had a known outcome and detailed clinical information available for analysis.

Description Ebola Virus Testing of Samples from Persons Under Investigation (*4*)

Extraction of RNA

Human blood samples or oral swab samples in AVL–carrier RNA buffer samples were first heat-inactivated at 62°C for 60 min. Then, viral RNA was extracted from 140 μL of sample by using QIAamp Viral RNA Mini Kit (QIAGEN, Germantown, MD, USA) according to the manufacturer's instructions and eluted in 60 μL of buffer. The negative and positive controls in the detection kit were also extracted to monitor the efficiency and inhibition of extraction and amplification.

Real-Time Reverse Transcription PCR (qRT-PCR)

Tests for the presence of Ebola virus were performed on site by using a qRT-PCR assay targeting the glycoprotein gene of Ebola virus subtype Zaire with primers 5′-TGGGCTGAAAAYTGCTACAATC-3′ (forward) and 5′-CTTTGTGMACATASCGGCAC-3′(reverse) and probe FAM-5′-CTACCAGCAGCGCCAGACGG–3′-TAMR) as previously described by Gibb et al. (*4*) and a 1-step qRT-PCR assay (Puruikang Biotech Co. Ltd, Shenzhen, China). RT-PCR was conducted by using the LightCycler 96 System (Roche Co. LTD, Basel, Switzerland) in a 25-μL mixture containing 3 μL of viral RNA, 20 μL of PCR buffer A, and 2 μL of PCR buffer B according to the manufacturer's instructions. The reaction was performed for 5 min at 42°C, followed by 10 s at 94°C, with a subsequent 40 cycles of amplification (94°C for 5 s, 55°C for 30 s). Fluorescence was recorded at 55°C.

A calibration standard was generated by diluting virus-like particles containing glycoprotein gene Zaire subtype Ebola virus based on the published sequence in GenBank. The dilutions (5×10^6 copies/mL) were subjected to RT-PCR analyses as a positive control test. The cycle threshold (CT) value of the positive control was used to estimate the absolute concentration

of the RNA, which was representative of the original virus load. The approximate virus load (copies/mL) of the original sample was calculated and converted as follows: $5\times10^6\times2^{(CT1-CT2)}$, where CT2 represents the CT value of samples and CT1 is the CT value of the diluting the positive control each test.

References

1. Schieffelin JS, Shaffer JG, Goba A, Gbakie M, Gire SK,Colubri A, et al. Clinical illness and outcomes in patients with Ebola in Sierra Leone. N Engl J Med. 2014;371:2092–100. PubMed http://dx.doi.org/10.1056/NEJMoa1411680

2. Wamala JF, Lukwago L, Malimbo M, Nguku P, Yoti Z, Musenero M, et al. Ebola hemorrhagic fever associated with novel virus strain, Uganda, 2007–2008. Emerg Infect Dis. 2010;16:1087–92. PubMed http://dx.doi.org/10.3201/eid1607.091525

3. WHO Ebola Response Team. Ebola virus disease in West Africa—the first 9 months of the epidemic and forward projections. N Engl J Med. 2014;371:1481–95. PubMed http://dx.doi.org/10.1056/NEJMoa1411100

4. Gibb TR, Norwood DA Jr, Woollen N, Henchal EA. Development and evaluation of a fluorogenic 5′ nuclease assay to detect and differentiate between Ebola virus subtypes Zaire and Sudan. J Clin Microbiol. 2001;39:4125–30. PubMed http://dx.doi.org/10.1128/JCM.39.11.4125-4130.2001
